# Adolescent Abstinence and Unprotected Sex in CyberSenga, an Internet-Based HIV Prevention Program: Randomized Clinical Trial of Efficacy

**DOI:** 10.1371/journal.pone.0070083

**Published:** 2013-08-14

**Authors:** Michele L. Ybarra, Sheana S. Bull, Tonya L. Prescott, Josephine D. Korchmaros, David R. Bangsberg, Julius P. Kiwanuka

**Affiliations:** 1 Center for Innovative Public Health Research, San Clemente, California, United States of America; 2 Department of Community and Behavioral Health, Colorado School of Public Health, Aurora, Colorado, United States of America; 3 Mbarara University of Science and Technology, Ragon Institute of Massachusetts General Hospital, Massachusetts Institute of Technology and Harvard, Massachusetts General Hospital Center for Global Health, Boston, Massachusetts, United States of America; 4 Department of Pediatrics, Mbarara University of Science and Technology, Mbarara, Uganda; Tulane University, United States of America

## Abstract

**Context:**

Cost-effective, scalable programs are urgently needed in countries deeply affected by HIV.

**Methods:**

This parallel-group RCT was conducted in four secondary schools in Mbarara, Uganda. Participants were 12 years and older, reported past-year computer or Internet use, and provided informed caregiver permission and youth assent. The intervention, CyberSenga, was a five-hour online healthy sexuality program. Half of the intervention group was further randomized to receive a booster at four-months post-intervention. The control arm received ‘treatment as usual’ (i.e., school-delivered sexuality programming). The main outcome measures were: 1) condom use and 2) abstinence in the past three months at six-months' post-intervention. Secondary outcomes were: 1) condom use and 2) abstinence at three-month's post-intervention; and 6-month outcomes by booster exposure. Analyses were intention to treat.

**Results:**

All 416 eligible youth were invited to participate, 88% (n = 366) of whom enrolled. Participants were randomized to the intervention (n = 183) or control (n = 183) arm; 91 intervention participants were further randomized to the booster. No statistically significant results were noted among the main outcomes. Among the secondary outcomes: At three-month follow-up, trends suggested that intervention participants (81%) were more likely to be abstinent than control participants (74%; p = 0.08), and this was particularly true among youth who were abstinent at baseline (88% vs. 77%; p = 0.02). At six-month follow-up, those in the booster group (80%) reported higher rates of abstinence than youth in the intervention, no booster (57%) and control (55%) groups (p = 0.15); they also reported lower rates of unprotected sex (5%) compared to youth in the intervention, no booster (24%) and control (21%) groups (p = 0.21) among youth sexually active at baseline.

**Conclusions:**

The CyberSenga program may affect HIV preventive behavior among abstinent youth in the short term and, with the booster, may also promote HIV preventive behavior among sexually active youth in the longer term.

**Trial Registration:**

NCT00906178.

## Introduction

Over the past five years, the HIV prevalence rate in Uganda has risen from 6.4% to 7.3% [1]. The reason for this upsurge is unknown, yet it reinvigorates the call for effective and accessible prevention programs – especially for young people, who are developing sexual practices that they may carry with them for the rest of their lives. Many adolescent behavioral trends are encouraging: Age at first sex is increasing and teen pregnancy is decreasing [2]. And yet, data suggest that rates of adolescent condom use may be decreasing [1], [2]. It certainly appears to be an uncommon behavior: Two-thirds of unmarried, sexually active adolescents 15–19 years of age report not using a condom at last sex [1]. This may in part be because of a lack of healthy sexuality education. Even though 76% of new HIV infections in Uganda are caused by heterosexual transmission [3], life skills-based HIV education is only available in 15% of Ugandan schools [3]. Cost effective, scalable programs that could be delivered in a school setting are urgently needed.

Anticipating ever-increasing Internet access [4], we developed and tested CyberSenga, a culturally relevant, Internet-based HIV prevention program for Ugandan secondary school students. Results of the randomized control trial (RCT) are reported here. The main outcome of interest was HIV preventive behavior, defined as 1) sexual abstinence; and 2) condom use during vaginal sex in the past three months at six-month follow-up. Indicators of HIV preventive behavior at three-month follow-up were secondary outcomes. As an additional secondary analysis, trends were examined for six-month outcomes across three groups: control participants, intervention-only participants, and intervention+booster participants.

## Materials and Methods

The protocol for this trial and CONSORT checklist are available as supporting information; see Checklist S1 and Protocol S1. This was a multi-school, parallel-group RCT with adaptive randomization (arms were balanced by biological sex and prior sexual experience), conducted in four Ugandan schools. The clinic trial registration number is: NCT00906178.

### Ethics statement

The research protocol was reviewed and approved by Chesapeake IRB in the United States and Mbarara University of Science and Technology Ethical Committee in Mbarara, Uganda. Both committees approved the consent process, including the consent language. Permission forms were available to caregivers both in English and Runyankole, the local language in Mbarara. Written informed permission was obtained from the caregivers of day students; and from the school principals, who are the legal caregiver proxies, for boarding students. Day students took the informed permission forms home to have them signed by their caregiver and then they returned the completed forms to the research assistants. Informed written assent was obtained from all youth participants.

### Participants

Participants were 12 years of age and older (M: 16.1 years, SD: 1.4 years) and enrolled in one of our four partner secondary schools in Mbarara, Uganda. Additional eligibility requirements included: having used a computer or the Internet at least once in the past year, not having been part of the Youth Advisory Council that beta tested the intervention during program development [5], caregiver informed permission, and youth informed assent. Partner schools were purposefully recruited to reflect a diversity of social class and religion: two schools were private, church-founded (non-denominational) all-boys schools; the third was a private, Muslim, mixed-sex school; and the fourth was a public, mixed-sex school.

### Study setting

Mbarara municipality has a population of 83,700 and is the seventh largest urban center in Uganda [6]. The greater district is mostly rural. Mbarara district's net secondary school enrollment rate in 2009 was higher than the national average (34.8% versus 23.8%) [6].

### Intervention and control group design

The *Senga* in central Uganda is the name given to the paternal aunt, considered responsible for advising girls as they come of age on issues related to the marital roles of a wife, including running a household and sexual health. The *Kojja* is the Senga equivalent for boys. CyberSenga was conceptualized to integrate these culturally salient symbols into an Internet-based HIV prevention program.

Intervention content and exercises were informed by Fisher et al.'s Information-Motivation-Behavior model of HIV preventive behavior [7], [8], [9], along with formative research in the target population [4], [5], [10], [11]. Evidence-based HIV interventions for adolescents also were consulted [12], [13], [14]. Five one-hour intervention modules and a one-hour review module were designed: 1) Information about HIV (e.g., what is HIV and how is it prevented); 2) Decision Making and Communication (e.g., steps to solving a problem; strategies for communicating your solution to others assertively); 3) Motivations to be healthy (e.g., reasons why adolescents choose to be abstinent versus to have sex); 4) How to use a condom to be healthy (e.g., demonstration of correct condom use; testimonials from people similar to the participants who used condoms); 5) Healthy relationships (e.g., components of healthy relationships; strategies to address coercive gifts); and 6) Review. The full program can be found at: www.cybersenga.com.

Based upon our formative work, we anticipated that computer skills would be low among participants, even though participants were required to have a minimum level of exposure to computers and the Internet. As such, each of the first three modules had an Introduction section that taught the necessary computers skills to navigate that particular module. For example, the first module showed youth how to move from one page to the next by clicking on the flashing arrow at the bottom of the page. The second module taught youth how to type text into a text box. The third module taught users how to navigate an exercise where they had to move pieces around to complete a puzzle.

Four different versions of the intervention were created so content could be tailored by biological sex (male or female) and self-reported prior sexual experience (sexually active or abstinent/secondary abstinent). All versions contained the same concepts, but were presented in different ways to increase the saliency and personal relevance. For example, during program development, the Community Advisory Board (e.g., a group of local, adult community members) and youth questioned why females (both abstinent and sexually active) and abstinent males needed to learn how to use condoms. Although they agreed that males who were sexually active could benefit from this information, they felt it unnecessary to teach everyone condom use skills. Given data showing condom use at first sex is a strong predictor of current condom use [15], [16], the male and female abstinent modules were tailored to not only present information about using condoms correctly, but also to address the reasons why they needed to know this information: They were told that even though they were not currently having sex, when they were older, they would be in a healthy relationship where they would be ready to have sex. Thus, it was important to learn how to use a condom now so that they would be prepared in the future. To address the question of why this information was important for females (abstinent and sexually active), module text suggested that, like other parts of a healthy relationship, knowing how to use a condom correctly takes two people. Consequently, women as well as men needed to learn how to use a condom. Although it was not necessary to have tailored text in the sexually active male module to address why it was important for them to know how to use condoms, the issue of motivation did need to be addressed. In Uganda, there is a belief among some people that you are not a ‘real man’ until you have had sex, and this does not necessarily connote ‘sex with a condom’. Text for males (abstinent and sexually active) presented a twist on this notion, suggesting that ‘real men’ used condoms every time they had sex.

The control arm was ‘treatment as usual’: Participants in the control arm received no programming or interaction beyond the HIV programming that was currently being offered at their school as part of their usual schedule of extracurricular activities (e.g., talks sponsored by The AIDS Support Organisation (TASO)).

### Procedures

The intervention was originally conceived to be a sequential, six-module program to be completed over the course of six weeks. The program needed to be administered in the first term (12 weeks in length) so that follow-up assessments could be concluded in the same school year. Based upon extensive pilot testing [5], we knew that recruitment and enrollment would take two weeks and the administration of the baseline survey, another two weeks. The first week and last two weeks of the term needed to be protected for student exams and other school administration responsibilities. This left five weeks for the intervention to be delivered. Therefore, during the planning phase and well before the RCT began, we decided to change the initial plan and deliver the review module as a booster session between the three-month and six-month follow-up surveys. Half of the intervention participants were randomly allocated to the booster and half were not.

Students were screened for eligibility and recruited in February 2011 and completed baseline surveys in March 2011, with intervention delivery occurring directly afterward. Three-month follow-up assessments were collected at the beginning of June 2011. The booster was delivered in July 2011. Six-month follow-up data were collected in September 2011. Baseline and follow-up surveys were completed online.

All surveys and CyberSenga intervention content were written in English, the official language of Uganda and language of instruction in schools (although a non-primary language for students) [17].

#### Recruitment

RCT participants were recruited in coordination with school staff and oversight from the principal investigator. Ideally, all youth would have been screened to establish eligibility (i.e., those who had used a computer or the Internet in the past year) and then participants would have been randomly selected from among the pool of eligible youth. Time constraints as discussed above made this infeasible, so instead, a subsample of randomly identified students was screened. Within each grade and for each school, the sample sizes of boys and girls that we needed to screen in order to identify a sufficient number of eligible youth, were based upon a previous survey conducted during the development phase of the project [11]. We thus used the previous estimates of participant response rates as well as the rate of Internet use reported by youth.

The headmaster of each school provided a current, alphabetical class list of students enrolled in Secondary 2–4 classes. After receiving the student roster list from the partner schools, the RAs data entered the student names from the class list in the order the names appeared, as well as their class level and biological sex. Youth were then randomly selected by the research team using randomizer.org. The resulting screening list was posted at each school the morning that screening was scheduled to take place.

At each school, screening was conducted simultaneously in multiple classrooms by several RAs, so that the process could be completed in one day. When students arrived to the classroom, the RAs first verified they were on the screening list and then gave them a screener to complete. Students placed their screener face down in a box at the front of the class room after they had completed it. In cases where the class/biological sex screening sample size was not met, screeners were left with the head teacher so they could attempt to screen students on the list who had been absent.

An online survey was created to capture the results (i.e., students that were eligible, ineligible, expelled, and did not show up for screening). The following day after screening a school, separate RAs double entered each of the screeners received.

#### Enrollment

A list of eligible youth was generated and provided to the RAs to direct their enrollment activities. RAs went to the schools in the afternoons, as classes were finishing for the day, to reach the identified youth. They explained the study and provided youth with the parent permission forms and youth assent forms, and explained that caregiver permission was required for them to participate. RAs returned the following several days to pick up the permission and assent forms. At least four attempts were made to enroll each eligible student.

#### Intervention delivery

Because two of the partner schools did not have Internet, computer access, or electricity in the classrooms, we created ‘mobile cafés’ to conduct the baseline and follow-up RCT assessments, and to deliver the intervention. For consistency across sites, we implemented the café in all four schools. Each day at the schools, RAs brought in ten netbooks (i.e., mini laptops) and an Internet router that was powered by a car battery.

Intervention participants began the CyberSenga program the week after the baseline surveys were completed. Intervention participants were scheduled to complete modules on specific weekdays after school hours (e.g., every Monday). RAs provided appointment reminder cards and actively sought out participants who did not show up for their scheduled sessions so that they could complete the module that day, or reschedule for another day. When participants showed up for their sessions, RAs directed them to one of the computers, and helped them log in to the CyberSenga system as needed. At the initial session, RAs provided a brief computer training course verbally for each youth. They then left participants to complete the CyberSenga modules independently, interrupting only when participants asked for assistance or they noticed participants having difficulty (e.g., Internet access problems; participants accidentally closing the CyberSenga program website). Confidentiality was ensured with privacy screens on every netbook. RAs were trained to answer student questions quietly so that those around them could not hear; and only to look at participant's computer screens if invited to do so by participants.

The CyberSenga system required users to click through each page of the module before they were allowed to advance to the next module, thus ensuring that participants viewed the session in full. Participants could revisit modules that they already completed but not skip ahead to future modules. The CyberSenga system tracked and guided participants' progress, and directed them back to where they left off in the module when they next logged in if they were unable to complete it in one sitting.

We endeavored to keep participants' exposure to the CyberSenga program modules as close to the desired program timeline (i.e., one module every seven days) as possible, but were flexible and allowed participants to complete the modules on an alternative schedule if necessary. As reported elsewhere [18], 95% of intervention participants completed all five modules.

#### Incentives

Participants did not receive incentives for their participation in the study. Each received a certificate of program completion as a thank you after completing the six-month follow-up survey.

### Sample size

Because of the novelty of this type of adolescent intervention, we were unable to identify any previous, empirically tested HIV prevention programs for secondary school students in Uganda. Consequently, the power analysis for this study was based upon the best available data sources available at the time of intervention planning: Based upon UNAIDS prevalence data [19], we predicted that 38% of males and 56% of females in our RCT would not have used a condom at last sex at baseline. With a target sample size of 300, we had 80% power (using alpha = 0.05) to detect an odds ratio of 1.43 or higher for control versus intervention participants reporting unprotected sex at six months. Prior to field, when the protocol was altered to accommodate the school schedule such that half of the intervention group would receive the booster and half would not, we also increased our target sample size to recruit 400 youth: 100 adolescents per school, 33 per class (50% of each class female in the mixed-sex schools). An equivalent sample size for each school was chosen so that the burden associated with the intervention delivery would be equal across school sites; and in so doing, also ensure that each school experienced minimal disruption.

### Randomization and masking

Randomization to the intervention or control arm was executed using code embedded in the software program that minimized imbalance between the study arms with respect to biological sex and prior sexual activity at baseline, while maintaining a ratio of 1∶1 in the two groups. Participants were randomized at the end of the baseline survey. As such, all participants were blind to their arm assignment at enrollment. Randomization to the booster session within the intervention group was applied at the end of the initial field period (i.e., after completion of the CyberSenga program). As with the initial procedure, randomization to the booster arm was stratified by biological sex and baseline sexual activity. Neither the research staff nor the participants were masked to study arm assignment.

#### Defining abstinent and sexually active

All youth needed to be coded as either sexually active or abstinent to enable the randomization code to work properly, and so that youth assigned to the intervention could be triaged to the appropriate version (i.e., abstinent or sexually active). Youth were coded based upon their responses to baseline questions about vaginal and anal sex: “Have you ever played vaginal sex? (We mean when a penis goes into a vagina)”; and “Have you ever played anal sex? (We mean when a penis goes into an anus)”. Participants who declined to answer either question were prompted to answer the relevant question with the following text: “We did not receive your answer for this question. Please enter your response. Remember that your answers are completely private, so please be honest.” Youth who did not provide an answer to the follow-up prompt (n = 4) were coded as abstinent in accordance with local norms and the wishes of our Community Advisory Board.

To further reflect local norms, youth who were not currently sexually active were coded as engaging in ‘secondary abstinence.’ These youth were treated as abstinent in the randomization code and pathing in the intervention. Secondary abstinence was defined as not having had sex in the past two years. Previous research has defined secondary abstinence as not having had sex in the past year [20], [21], [22]. Acknowledging that sometimes youth are abstinent not by choice but because of lack of opportunity, we chose to be conservative in our definition.

To identify youth engaging in secondary abstinence at baseline, those who reported having had vaginal or anal sex ever were asked: “When was the last time you played vaginal [anal] sex with your current (or most recent) sexual partner?” Response options were: In the last 1 month; More than one month but less than three months ago; three months or more but less than six months ago; six months or more but less than 12 months ago; 12 months or more but less than 24 months (two years) ago; 24 months (two years) ago or longer; and Do not want to answer. Youth who reported having had sex more than 24 months (two years ago) were deemed to be engaging in ‘secondary abstinence’ and coded as abstinent. Youth who did not want to answer (n = 6) also were coded as abstinent.

### Outcomes

The study design initially proposed to examine the effects of exposure to CyberSenga on unprotected sex over the six-month follow-up period. Based upon the decision to deliver the final module as a booster, the main outcome measure was modified, prior to study implementation, to be unprotected sex in the past three months at six-months' post-intervention. This allowed us to identify potential changes in rates of HIV preventive behavior over time (e.g., attenuation of effect over time; difference in effect between those in the booster versus not once it had been administered).

Recognizing that the majority of participants would likely be sexually abstinent at baseline [11], we also reclassified abstinence from a secondary to a main outcome.

Abstinence was defined as not having had vaginal or anal sex in the past three months at follow-up. Sex was queried with two questions using culturally understandable terminology: 1) “Have you played vaginal sex since you did the CyberSenga survey in first term in February *[second term in June]* (about three months ago)? (We mean when a penis goes into a vagina)”; and 2) “Have you played anal sex since you did the CyberSenga survey in first term in February *[second term in June]* (about three months ago)? (We mean when a penis goes into an anus).”

Unprotected vaginal sex was measured by asking participants who reported vaginal sex in the past three months, first, how many times they had had vaginal sex in the past three months and then second, how many times they had used a condom when having vaginal sex in the past three months. Youth who reported anything less than 100% condom use were coded as having had unprotected vaginal sex.

### Statistical analyses

Except for outcome variables, non-responsive answers (i.e., ‘decline to answer’) to survey items included in the analyses were imputed using multiple imputation techniques [23]. In most cases, variables had less than five percent of data imputed.

The effectiveness of the randomization was examined by comparing youth characteristics in the control versus intervention groups using chi-square tests. Next, the influence of the intervention on behavioral outcomes was tested. The relative odds of abstinence and unprotected sex were each estimated given intervention versus control group assignment. Models were adjusted for predictors of sexual activity: sex, age, social support from a special person, and HIV preventive motivation. Analyses were intent-to-treat (ITT) (i.e., all randomized individuals were included in the analysis) and per-protocol to provide a type of sensitivity analysis (i.e., showing the influence that coding missing values to ‘sexually active’ had on the findings). Analyses were reported for all youth and also stratified by baseline sexual behavior. Finally, questions that assessed exposure to the intervention were examined for differences in accuracy between intervention and control participants to determine whether contamination between the two experimental groups had occurred.

## Results

### Recruitment

Based upon prevalence rates of computer use in a previous survey [11], we aimed to screen all female students enrolled in the two mixed-sex partner schools (n = 382) and 772 of the 2,264 male students enrolled across the four partner schools in order to identify 400 eligible youth for the RCT. Because the screeners were administered the second week of the first school term, many youth were out of school trying to secure school fees. Also, some youth had changed schools since the list had been created. As a result, 740 of the 1,154 identified youth were screened, 416 of whom were eligible (56% of those screened). The sole reason for being ineligible was not having computer or Internet exposure in the past year.

**Figure 1:**
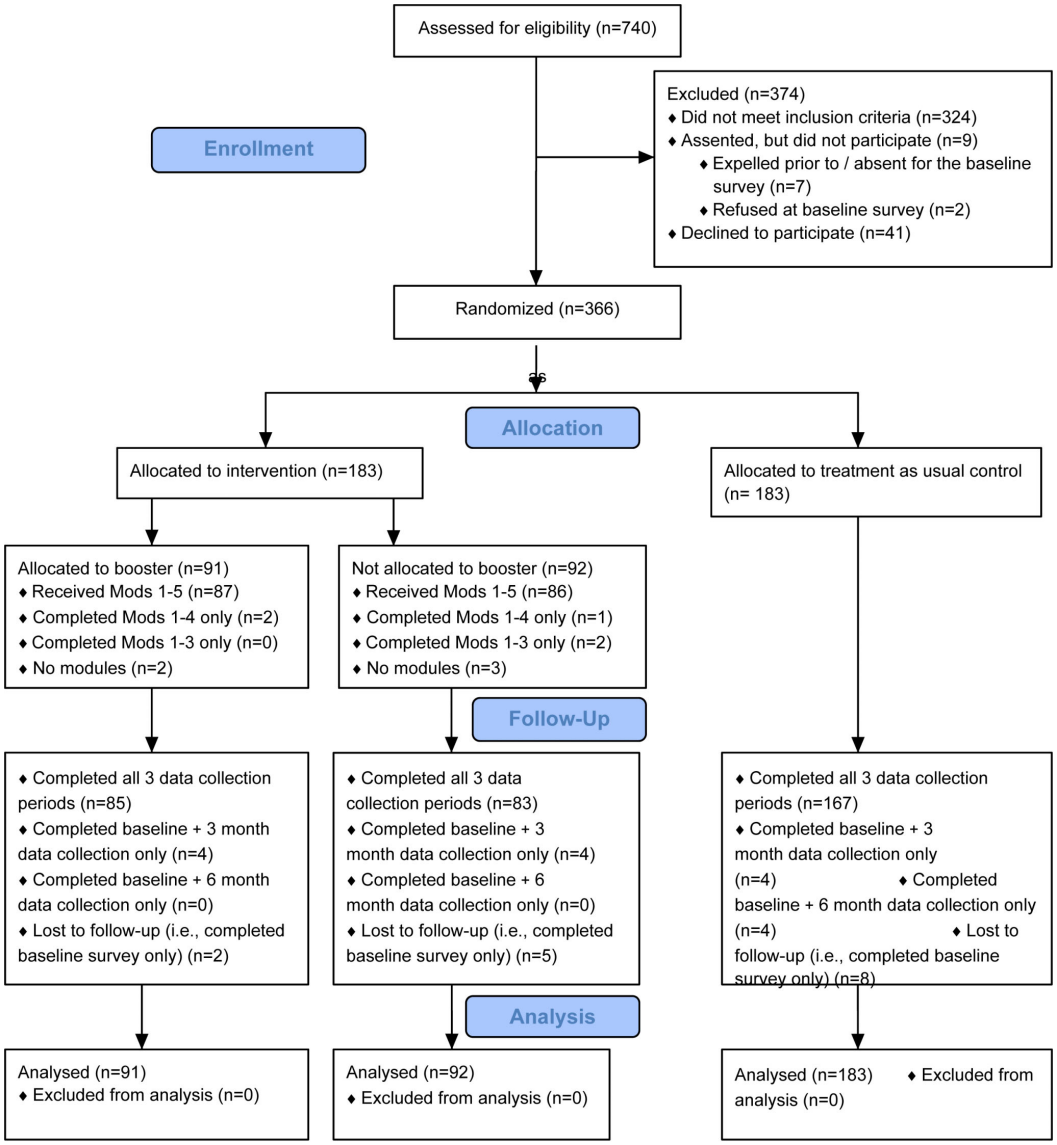
CyberSenga Randomized Controlled Trial Consort Diagram.

Eighty-eight percent (n = 366) of eligible students provided signed adult permission and youth assent forms, completed the baseline survey, and were randomized (see Figure 1). Of the 12% (n = 50) who were eligible but did not participate: two caregivers declined to provide permission and thirty-nine youth declined to provide assent; two youth provided assent but then declined at the baseline survey, and seven provided assent but were not present at the time of the baseline surveys (these youth were given multiple chances to complete the survey over the two-week field period).

The rates of youth screened and deemed to be eligible differed by school, and reflected the relative differences in socioeconomic status (SES) of the schools' students (Table 1). For example, in lower SES schools, more youth were absent during screening because they were gathering school fees. Youth in lower SES schools also were less likely to report computer/Internet experience. Enrollment rates among eligible youth were similar across schools, however. Additionally, similar rates of female (60%) and male (66%) students were successfully screened, although less than half as many females (29%) than males (68%) were eligible. Among those who were screened and eligible, 88% of males and females, respectively, were enrolled.

**Table 1:** Participant characteristics across the four schools (n = 366).

Participant characteristics	School 1	School 2	School 3	School 4
	%	%	%	%
Recruitment characteristics				
Screened	79.1%	55.0%	76.5%	64.7%
Eligible	77.8%	40.1%	91.4%	45.5%
Enrolled	89.3%	83.9%	89.5%	89.7%
Demographic characteristics				
Age (M: SD)	15.8 (1.4)	16.5 (1.5)	15.7 (1.3)	16.3 (1.5)
Female	0.0%	30.9%	0.0%	34.5%
Grade				
Secondary 2	29.0%	27.7%	29.4%	19.5%
Secondary 3	35.0%	30.9%	32.9%	44.8%
Secondary 4	36.0%	41.5%	37.7%	35.6%
Day student	0.0%	93.6%	0.0%	12.6%
Maternal schooling primary school or less, or don't know	31.0%	44.7%	31.8%	26.4%
Paternal schooling primary school or less, or don't know	20.0%	37.2%	27.1%	23.0%
Infrequent Internet use (monthly or less)	37.0%	81.9%	42.4%	60.9%
HIV indicators				
Ever been tested for HIV	35.0%	38.3%	36.5%	42.5%
Known someone who has died of AIDS	40.0%	41.5%	30.6%	37.9%
Tired of hearing about HIV prevention information (somewhat/strongly agree)	17.0%	29.8%	18.8%	37.9%
Above average chance of getting HIV	6.0%	10.6%	4.7%	4.6%
Answered at least 80% of the HIV information questions accurately	58.0%	42.6%	56.5%	42.5%
Dating and sexual behavior				
Ever had a boyfriend or girlfriend	88.0%	70.2%	69.4%	74.7%
Ever been a victim of dating violence (all youth)*	19.0%	33.0%	11.8%	20.7%
Ever been a perpetrator of dating violence (all youth) *	15.0%	28.7%	9.4%	17.2%
Ever had oral sex	9.0%	7.4%	11.8%	11.5%
Ever had vaginal sex	37.0%	26.6%	30.6%	28.7%
Ever had anal sex	0.0%	1.1%	5.9%	3.4%
Somatic/psychosocial indicators				
Wish to have more self-respect	83.0%	78.7%	77.6%	80.5%
Fair or poor health	12.0%	14.9%	27.1%	11.5%
Bright future' somewhat/very unlikely	7.0%	10.6%	9.4%	11.5%

* Rates are shown of all youth to provide a population-based estimate of dating violence involvement. Data are confounded by the rate of youth who have ever had a boyfriend or girlfriend.

Samples sizes by school are not shown to protect the identity of each school.

### Baseline data

The intervention and control arms were well balanced on almost all youth characteristics: Boarding versus day-scholars and family social support were exceptions (Table 1).

Thirty-one percent of youth (n = 114) reported ever having vaginal or anal sex; 23% (n = 83) in the past two years. Half of youth who had ever had vaginal or anal sex (n = 57) reported using and half reported not using (n = 57) a condom the last time they had sex. The median age of first sex was 14 years (Mean: 13.7 years, SD: 2.4 years; Range: 10 years or younger – 19 years of age or older). Additional sample characteristics are shown in Table 2.

**Table 2:** Participant sample characteristics by arm assignment (n = 366).

	All Youth (n = 366)	Arm assignment	
Participant characteristics		Control group (n = 183)	Intervention group (n = 183)
	% (n)	% (n)	% (n)
Demographic characteristics			
Age (M: SD; Range: 13–19+ years)	16.1 (1.4)	16.2 (1.5)	16.0 (1.4)
Female	16.1% (59)	15.3% (28)	16.9% (31)
Grade			
Secondary 2	26.5% (97)	27.3% (50)	25.7% (47)
Secondary 3	35.8% (131)	31.7% (58)	39.9% (73)
Secondary 4	37.7% (138)	41.0% (75)	34.4% (63)
Day scholar	27.3% (100)	22.4% (41)	32.2% (59)
Maternal education primary school or less/don't know	33.6% (123)	34.4% (63)	32.8% (60)
Paternal education primary school or less/don't know	26.8% (98)	27.3% (50)	26.2% (48)
Infrequent Internet use (monthly or less)	55.5% (203)	55.7% (102)	55.2% (101)
History of sexual behavior			
Ever had oral sex	9.8% (36)	9.3% (17)	10.4% (19)
Ever had vaginal sex	30.9% (113)	30.0% (55)	31.7% (58)
Ever had anal sex	2.5% (9)	2.7% (5)	2.2% (4)
HIV-related experiences and beliefs			
Ever been tested for HIV	38.0% (139)	35.0% (64)	41.0% (75)
Ever known someone who died from AIDS	37.7% (138)	38.8% (71)	36.6% (67)
Tired of hearing about HIV prevention information (somewhat/strongly agree)	25.7% (94)	26.8% (49)	24.6% (45)
Above average chance of getting HIV (self-appraised)	6.6% (24)	6.6% (12)	6.6% (12)
Beliefs supportive of HIV stigma (M: SD; Range: 0–4)	1.1 (1.0)	1.2 (1.1)	1.1 (0.9)
Romantic relationships			
Ever had a boyfriend/girlfriend	76.0% (278)	74.9% (137)	77.0% (141)
Ever been a victim of teen dating violence	21.3% (78)	23.5% (43)	19.1% (35)
Ever been a perpetrator of teen dating violence	17.8% (65)	20.2% (37)	15.3% (28)
Beliefs consistent with female empowerment in relationships (M: SD; Range: 2–10)	8.1 (2.8)	8.0 (2.8)	8.2 (2.8)
Somatic/psychosocial health indicators			
Fair or poor health	16.1% (59)	13.1% (24)	19.1% (35)
Bright future' somewhat/very unlikely	9.6% (35)	8.2% (15)	10.9% (20)
Wish to have more self-respect	80.0% (293)	78.7% (144)	81.4% (149)
Social support from a special person (M: SD; Range: 4–20)	16.5 (4.0)	16.2 (4.1)	16.7 (3.8)
Social support from family (M: SD; Range: 4–20)	17.4 (3.1)	17.7 (3.0)	17.1 (3.1)
Information-Motivation-Behavior Model constructs			
Information: 80% or more answers about HIV correct	50.0% (183)	53.0% (97)	47.0% (86)
Motivation: Attitudes towards HIV preventive acts (M: SD; Range: 1–5)	3.6 (0.9)	3.5 (0.9)	3.6 (0.9)
Motivation: Subjective norms regarding HIV preventive acts (M: SD; Range: 1–5)	3.6 (0.9)	3.5 (0.9)	3.6 (0.9)
Motivation: Behavioral intentions for HIV prevention (M: SD; Range: 1–5)	3.5 (0.9)	3.5 (0.9)	3.5 (0.9)
Behavioral skills (M: SD; Range: 1–5)	2.9 (0.7)	2.9 (0.7)	2.9 (0.7)

Youth who had had vaginal or anal sex in the past two years were significantly (p<0.01) older, more likely to be male, have had a boyfriend or girlfriend as well as have been involved in teen dating violence as a victim and/or perpetrator, have higher support from a special person, and lower scores of all three types of HIV preventive motivation. Across schools, youth significantly differed (p<0.01) by age, frequency of Internet use, HIV prevention information fatigue, having a boyfriend or girlfriend and being involved in dating violence as a victim and/or perpetrator, as well as characteristics typifying the different schools (i.e., biological sex, boarding versus day school; Table 1). Rates of vaginal sex were not statistically different across the schools, although rates were somewhat higher at the all-boys, boarding schools.

Of the 307 male study participants, 36% (n = 110) reported ever having vaginal or anal sex (1% (n = 4) declined to answer), 73% (n = 80) of whom reported having sex in the past two-years. Of the 59 female study participants, 7% (n = 4) reported ever having had vaginal or anal sex (0% n = 0) declined to answer), 75% (n = 3) of whom reported having had sex in the past two-years. Consequently, 62% (n = 227) of participants were categorized as abstinent males, 15% (n = 56) as abstinent females, 22% (n = 80) as sexually active males, and 1% (n = 3) as sexually active females. Of the 183 intervention participants, 61% (n = 112) were assigned to the male abstinent CyberSenga program, 22% (n = 40) to the male sexually active program, 16% (n = 30) to the female abstinent program, and <1% (n = 1) to the female sexually active program.

### RCT outcomes: Program retention

Ninety-six percent of intervention and 93% of control participants provided three-month follow-up data (χ^2^(1) = 1.4, p = 0.24). Ninety-two percent of intervention and 93% of control participants provided six-month follow-up data (χ^2^(1) = 0.4, p = 0.55). All 366 randomized youth were included in the intention-to-treat analyses; youth lost to follow-up were assumed to have had unprotected sex and not be abstinent. Three hundred and forty-seven and 339 youth were included in the per-protocol analyses at three-month and six-month follow-ups, respectively. Reported analyses of the entire sample were pre-specified; subgroup analyses (e.g., among youth abstinent at baseline) were exploratory. No harm to any participant was noted.

### RCT main outcomes: Abstinence and unprotected sex at six-month follow-up

Among all youth, 81% (n = 183) of abstinent males (i.e., those who had never had sex, or had sex no more recently than two years ago) at baseline reported being abstinent in the past three months at six-month follow-up. Eighty-six percent (n = 48) of abstinent females also reported past-three-month abstinence at six-month follow-up. Among sexually active youth, 29% (n = 23) of males and 0% (n = 0) of females reported past-three-month vaginal sexual activity at six-month follow-up (Table 3).

**Table 3:** Sexual behavior at three- and six-month follow-up based upon baseline behavior by arm assignment (n = 366).

		Control (n = 155)						Intervention (n = 152)					
Males		Abstinent at baseline (n = 115)			Sexually active at baseline (n = 40)			Abstinent at baseline (n = 112)			Sexually active at baseline (n = 40)		
		Abstinent at follow-up	Vaginal sex in the past three-months at follow-up	Non-responder or declined to answer at follow-up	Abstinent at follow-up	Vaginal sex in the past three-months at follow-up	Non-responder or declined to answer at follow-up	Abstinent at follow-up	Vaginal sex in the past three-months at follow-up	Non-responder or declined to answer at follow-up	Abstinent at follow-up	Vaginal sex in the past three-months at follow-up	Non-responder or declined to answer at follow-up
	Three-month follow-up	75.6% (87)	14.8% (17)	9.6% (11)	62.5% (25)	37.5% (15)	0.0% (0)	85.7% (96)	9.8% (11)	4.5% (5)	60.0% (24)	37.5% (15)	2.5% (1)
	Six-month follow-up	81.7% (94)	12.2% (14)	6.1% (7)	57.5% (23)	35.0% (14)	7.5% (3)	79.5% (89)	13.4% (15)	7.1% (8)	70.0% (28)	22.5% (9)	7.5% (3)

		Control (n = 28)						Intervention (n = 31)					
Females		Abstinent at baseline (n = 26)			Sexually active at baseline (n = 2)			Abstinent at baseline (n = 30)			Sexually active at baseline (n = 1)		
		Abstinent at follow-up	Vaginal sex in the past three-months at follow-up	Non-responder or declined to answer at follow-up	Abstinent at follow-up	Vaginal sex in the past three-months at follow-up	Non-responder or declined to answer at follow-up	Abstinent at follow-up	Vaginal sex in the past three-months at follow-up	Non-responder or declined to answer at follow-up	Abstinent at follow-up	Vaginal sex in the past three-months at follow-up	Non-responder or declined to answer at follow-up
	Three-month follow-up	92.3% (24)	3.8% (1)	3.8% (1)	100.0% (2)	0.0% (0)	0.0% (0)	96.7% (29)	0.0% (0)	3.3% (1)	100.0% (1)	0.0% (0)	0.0% (0)
	Six-month follow-up	88.5% (23)	3.8% (1)	7.7% (2)	100.0% (2)	0.0% (0)	0.0% (0)	83.3% (25)	3.3% (1)	13.3% (4)	100.0% (1)	0.0% (0)	0.0% (0)

#### Abstinence

Based upon intention-to-treat, past-three-month abstinence rates were similar for control (75%) and intervention (75%) participants at six-month follow-up (p = 0.90). Findings were similar per-protocol. No other comparisons were statistically significant (see Table 4, Model 1).

**Table 4:** Past three-month abstinence at follow-up among adolescents in the CyberSenga intervention (n = 366).

Main Outcome: Six-month follow-up	Model 1: Intervention versus control			Model 3: Intervention and Intervention+Booster versus Control				
	Control (n = 183)	Intervention (n = 183)	aOR	Control* (n = 183)	Intervention, nb (n = 92)	aOR	Intervention +b (n = 91)	aOR
	% (n)	% (n)	(95% CI)	% (n)	% (n)	(95% CI)		(95% CI)
Intention to treat (n = 366)								
All youth (n = 366)	74.9% (137)	75.4% (138)	1.01 (0.62, 1.65)	74.9% (137)	73.9% (68)	0.96 (0.53, 1.72)	76.9% (70)	1.07 (0.58, 1.97)
Abstinent youth at baseline (n = 283)	80.9% (114)	77.5% (110)	0.79 (0.44, 1.43)	80.9% (114)	78.9% (56)	0.91 (0.44, 1.90)	76.1% (54)	0.68 (0.34, 1.40)
Sexually active youth at baseline (n = 83)	54.8% (23)	68.3% (28)	1.75 (0.67, 4.58)	54.8% (23)	57.1% (12)	1.03 (0.33, 3.29)	*80.0*% *(16)*	*3.22 (0.87, 11.91)*
Per-protocol (n = 339)								
All youth (n = 339)	81.9% (140)	84.5% (142)	1.15 (0.64, 2.08)	81.9% (140)	84.3% (70)	1.19 (0.57, 2.45)	84.7% (72)	1.12 (0.54, 2.33)
Abstinent youth at baseline (n = 262)	87.9% (116)	86.9% (113)	0.86 (0.40, 1.83)	87.9% (116)	89.1% (57)	1.13 (0.43, 2.97)	84.9% (56)	0.67 (0.27, 1.64)
Sexually active youth at baseline (n = 77)	61.5% (24)	76.3% (29)	2.13 (0.74, 6.11)	61.5% (24)	68.4% (13)	1.46 (0.41, 5.17)	*84.2*% *(16)*	*3.36 (0.78, 14.40)*

	Model 2: Intervention versus control		
Secondary Outcome: Three-month follow-up	Control (n = 183)	Intervention (n = 183)	aOR
	% (n)	% (n)	(95% CI)
Intention to treat (n = 366)			
All youth (n = 366)	*73.8% (135)*	*81.4% (149)*	*1.56 (0.93, 2.62)*
Abstinent youth at baseline (n = 283)	**77.3% (109)**	**88.0% (125)**	**2.27 (1.17, 4.39)**
Sexually active youth at baseline (n = 83)	61.9% (26)	58.5% (24)	0.73 (0.28, 1.88)
Per-protocol (n = 347)			
All youth (n = 347)	80.7% (138)	84.7% (149)	1.31 (0.73, 2.34)
Abstinent youth at baseline (n = 265)	86.0% (111)	91.9% (125)	1.97 (0.87, 4.45)
Sexually active youth at baseline (n = 82)	64.3% (27)	60.0% (24)	0.70 (0.27, 1.84)

aOR = adjusted odds ratio.

Intervention, nb = Intervention, no booster.

Intervetion +b = Intervention with booster.

Bold denotes statistically significant difference between the intervention and control arms at p≤.05.

Abstinent youth include youth who have never had sex, as well as those who have had sex, but not more recently than 2 years ago.

Italics denotes a suggestion of a difference between the intervention and control arms at p≥.05 but p≤.10.

* Reference group.

Odds ratios adjusted for: youth age, history of a boyfriend or girlfriend, support from a special person, Attitudes towards HIV preventive acts, Subjective Norms Regarding HIV Preventive acts, and Behavioral Intentions for HIV Prevention. Models for All Youth also are adjusted for biological sex; analyses stratified by baseline sexual experience are not due to collinearity.

#### Unprotected sex

Rates of past-three-month unprotected sex for the control (13%) and intervention (14%) groups were similar at six-month follow-up (p = 0.76). No other comparisons were statistically significant (see Table 5, Model 1).

**Table 5:** Percent of youth reporting unprotected vaginal sex in the past three months at three-month and six-month follow-up among adolescents in the CyberSenga intervention (n = 366).

Main Outcome: Six-month follow-up	Model 1: Intervention versus control			Model 3: Intervention and Intervention+Booster versus Control				
	Control (n = 183)	Intervention (n = 183)	aOR	Control* (n = 183)	Intervention, nb (n = 92)	aOR	Intervention +b (n = 91)	aOR
	% (n)	% (n)	(95% CI)	% (n)	% (n)	(95% CI)		(95% CI)
Intention to treat (n = 366)	% (n)	% (n)		% (n)	% (n)			
All youth (n = 366)	13.1% (24)	14.2% (26)	1.05 (0.57, 1.94)	13.1% (24)	16.3% (15)	1.20 (0.59, 2.45)	12.1% (11)	0.90 (0.41, 1.98)
Abstinent youth at baseline (n = 283)	10.6% (15)	14.1% (20)	1.32 (0.64, 2.72)	10.6% (15)	14.1% (10)	1.25 (0.52, 2.98)	14.1% (10)	1.40 (0.58, 3.36)
Sexually active youth at baseline (n = 83)	21.4% (9)	14.6% (6)	0.62 (0.18, 2.10)	21.4% (9)	23.8% (5)	1.51 (0.38, 6.03)	5.0% (1)	0.15 (0.02, 1.35)
Per-protocol (n = 339)								
All youth (n = 339)	7.0% (12)	6.6% (11)	0.86 (0.36, 2.08)	7.0% (12)	7.2% (6)	0.95 (0.33, 2.69)	5.9% (5)	0.78 (0.25, 2.39)
Abstinent youth at baseline (n = 262)	4.6% (6)	6.2% (8)	1.23 (0.40, 3.72)	4.6% (6)	4.7% (3)	0.92 (0.22, 3.88)	7.6% (5)	1.55 (0.44, 5.50)
Sexually active youth at baseline (n = 77)	15.4% (6)	7.9% (3)	0.38 (0.08, 1.84)	15.4% (6)	15.8% (3)	1.15 (0.22, 6.17)	0.0% (0)	NC

	Model 2: Intervention versus control		
Secondary Outcome: Three-month follow-up	Control (n = 183)	Intervention (n = 183)	aOR
	% (n)	% (n)	(95% CI)
Intention to treat (n = 366)			
All youth (n = 366)	15.3% (28)	11.5% (21)	0.70 (0.37, 1.30)
Abstinent youth at baseline (n = 283)	14.2% (20)	9.2% (13)	0.58 (0.27, 1.23)
Sexually active youth at baseline (n = 83)	19.0% (8)	19.5% (8)	1.06 (0.33, 3.44)
Per-protocol (n = 347)			
All youth (n = 347)	9.4% (16)	8.0% (14)	0.78 (0.36, 1.67)
Abstinent youth at baseline (n = 265)	6.2% (8)	5.2% (7)	0.80 (0.27, 2.34)
Sexually active youth at baseline (n = 82)	19.0% (8)	17.5% (7)	0.84 (0.24, 2.91)

aOR = adjusted odds ratio.

Intervention, nb = Intervention, no booster.

Intervetion +b = Intervention with booster.

NC = Not calculated (no observations).

Abstinent youth include youth who have never had sex, as well as those who have had sex, but not more recently than 2 years ago.

* Reference group.

Odds ratios adjusted for: youth age, history of a boyfriend or girlfriend, support from a special person, Attitudes towards HIV preventive acts, Subjective norms regarding HIV preventive acts, and Behavioral intentions for HIV prevention. Models for All Youth (except three-month follow-up per-protocol) also are adjusted for biological sex; analyses stratified by baseline sexual experience are not due to collinearity.

### RCT secondary outcomes

#### Abstinence at three-month follow-up

Trends suggested that intervention participants (81%) were more likely than control participants (74%) to be abstinent (p = 0.08) at three-month follow-up, and this was particularly true for youth who were abstinent at baseline: 88% of abstinent intervention versus 77% of abstinent control group participants reported past-three-month abstinence (p = 0.02). Indeed, adjusting for age, history of a romantic partner, support from a special person, and HIV prevention motivation, abstinent youth at baseline in the intervention were more than twice as likely to be abstinent at three months compared to their control group counterparts (aOR = 2.3, p = 0.015; Table 4, Model 2).

#### Unprotected sex at three-month follow-up

No notable findings for unprotected sex at three-month follow-up were observed.

#### Trends for the booster group at six-month follow-up

As shown in Table 4, Model 3, among youth who were sexually active at baseline, trends suggested that youth in the intervention+booster group (80%) were more likely than those in the control group (55%) to be abstinent in the past three months at six-month follow-up (aOR = 3.2; p = 0.08). Similarly non-significant, but promising trends were noted for unprotected sex: one-quarter as many youth in the intervention+booster group (5%) reported unprotected sex as those in the control group (21%; aOR = 0.15, p = 0.09; Table 5, Model 3).

### Contamination

As shown in Table 6, the intervention group was significantly more likely to correctly answer at least three of the four topics queried related to the program content (61%) than control participants (14%; p<0.001). Not only were they more likely to correctly identify concepts discussed in the program (e.g., that abstinent youth need to learn about condoms) but also program components (e.g., the ‘lion’, ‘lamb’ and ‘you’ in the Communication module).

**Table 6:** Indications of contamination: A comparison of the frequency of correct answers in the intervention and control groups to questions about the CyberSenga program content at three-month follow-up (n = 347).

Question about the CyberSenga program content	Control group (n = 171)	Intervention group (n = 176)	
	%(n)	%(n)	p-value
For teenagers, are there more good things about being abstinent or about playing sex	67.2% (115)	77.3% (136)	0.04
For teenagers, are there more bad things about being abstinent or about playing sex	67.2% (115)	65.3% (115)	0.71
If you accept a gift from someone and they demand sex, do you have to play sex with them - even if you do not want to	91.2% (156)	84.7% (149)	0.06
Is it true or false that teenagers who are abstinent do not need to know how to use condoms	70.2% (120)	81.8% (144)	0.01
What do the ‘lion’, ‘lamb’, and ‘you’ refer to	12.9% (22)	33.0% (58)	<0.001
Correctly identified at least 3 of the 4 topics of possible 8 that were included in the CyberSenga project	14.0% (24)	61.4% (108)	<0.001

## Discussion

This study is the first we are aware of to develop and test the feasibility and acceptability of an Internet-based HIV prevention program for adolescents in sub-Saharan Africa. The main outcomes of reduced unprotected sex and sustained abstinence at six-months were not supported. Findings nonetheless suggest that the CyberSenga program is associated with sustained abstinence among abstinent youth in the short term, and, when a booster is used, may also promote secondary abstinence and unprotected sex among sexually active youth in the longer term.

Even though intervention and control participants were within the same schools and attended classes side by side, data do not suggest that contamination was a significant problem. These findings add to the emerging literature indicating that contamination may not need to be a major concern [24]. Indeed, if contamination were a source of behavior change, it would make program dissemination much easier.

Eligibility requirements were minimal. As such, the intervention was implemented in a sample that was diverse in terms of age, biological sex, social class, sexual experience, and computer experience. Even though youth were required to have used a computer or the Internet in the past year, many lacked basic skills (e.g., use of the space bar to create a space in between words when typing). Findings then, are likely generalizable to the larger population of secondary school students in Uganda. The requirement that users have experience with computers could be removed and the CyberSenga program could be used as a universal HIV prevention program for Ugandan adolescents wherever Internet access is available. This may be particularly important for females. In contrast to the CyberSenga cohort, adolescent females tend to report higher rates of sexual activity than males [3], [25]. Although it is unclear why our sample was different, it may in part be because school is a protective factor against sex for females [1], [26], [27]. Future studies should focus on recruiting sexually active females from a diversity of environments to better examine whether CyberSenga has a differential impact by biological sex. Because females reported rates of computer and Internet exposure at less than half those of males, lifting the requirement for computer or Internet use could facilitate this.

Certainly, increased condom use is critical to reducing HIV and other sexually transmitted infections for young people who are having vaginal and anal sex. At the same time, abstinence is the most effective way to protect against these negative outcomes. Sexual activity in adolescence is not a simple issue however. Youth who are in relationships with older partners and abusive partners are more likely to have sex at an earlier age [28]. Poverty and social norms also play a part [29]. The decision whether or not to have sex is not a simple ‘yes’ or ‘no’ therefore, but is made within the context of many other factors that a young person must successfully navigate. CyberSenga is a comprehensive sexuality program that acknowledges these complex factors. For example, the disproportionate power inherent in relationships with older partners and the potential health risks of having sex for economic gain are presented. Intervention content discusses the benefits and drawbacks of being abstinent and compares them to the benefits and drawbacks of having sex as an adolescent. Current findings provide further support for the hypothesis that abstinent behavior can be affected in comprehensive sexuality programs that address cultural as well as sexual health issues, and that such programs can have the added benefit of also affecting condom use among those who are choosing to have sex. This is critical in settings with high HIV prevalence, where the potential consequences of having sex as an adolescent are much greater.

Many HIV prevention programs focus solely on sexually active youth as they are the ones at immediate risk for contracting sexually transmitted infections. Given that current condom use is most strongly predicted by condom use at first sex [15], [16] however, a strength of the CyberSenga program is that it targets both sexually active youth (who are at greater risk for HIV) and abstinent youth (who will become sexually active at some point in their lives). This heterogeneity of sexual experience poses both practical and analytical challenges, mostly due to recruiting sufficient numbers of youth in each group and identifying outcomes that are likely to change over the observation period. Despite these challenges, it is critical in settings with high HIV burden, such as Uganda, to develop and test prevention content that is relevant to both sexually active and sexually abstinent youth so that youth of all sexual experience levels can have the tools necessary for safe sexual experiences.

Relatedly, pre-study estimates of non-condom using, currently sexually active youth (38% of males and 56% of females) were higher than what was self-reported in this younger, school-attending sample of adolescents. The number of youth classified as sexually active was further reduced by our decision to treat participants who had not had sex in the past two years as abstinent. The wide divergence between the power analysis assumptions and the actual study sample experience likely affected our ability to detect statistically significant differences. The disparity between projected and actual sexual activity rates supports the need for smaller scale studies first to inform more accurate power estimates once feasibility is demonstrated. Next steps for the current research include implementing a larger, fully-powered trial based upon accurate effect size estimates generated from this trial.

The study included a wide age range across adolescence. This had the potential to result in developmentally normative differences within the intervention group in terms of youth's experiences with romantic relationships, the quality and intensity of these relationships, and motivations for sex. In Uganda, there are few freedoms gained with increased age among adolescents however. For example, it is against secondary school policy for youth of *any* age to be in a dating relationship. Furthermore, none of the students had a car or a driver's license. The concept of having sex in a car or being able to drive to a private place to have sex was inconceivable. Across all age groups, youths' movement were tightly constricted in both home and school environments. Accordingly, age-specific scenarios of sexual encounters did not seem to emerge during the qualitative formative work done with adolescents. Instead, an age-transcendent narrative of hurried pressure to have sex when youth found themselves unexpectedly alone (e.g., in a classroom after school hours, on a walk, etc.) was commonly voiced. When the program is disseminated to other settings, it will be important to explore whether tailoring content to discuss age-specific scenarios is needed.

The infrastructure in some of the schools required extensive work-arounds to ensure electricity, Internet, and computer access. We learned that a lack of or variable electricity can be overcome with battery-powered netbooks and car batteries (or perhaps solar power in the future) to power Internet routers. Whether the intervention can feasibly be implemented in the future without these extra resources is unknown.

Additional limitations merit discussion. Sexual activity in adolescence is a stigmatized behavior in Uganda, especially for females. Youth may have under-reported their sexual experiences, which in the intervention group would have led to them being triaged to the incorrect content. It also may be possible that exposure to the intervention may have affected this social desirability bias, either by making youth more comfortable with their sexual experiences so that they were more likely to honestly report sexual activity than the control group at follow-up; or by further reinforcing the importance of abstinence in adolescence such that they would be less likely to honestly report sexual activity than the control group. It also needs to be noted that CyberSenga is relevant for an important population of young people, but it is not designed for nor will it reach *all* youth. Certainly, with 24% of youth enrolled in secondary schools [6], additional intervention efforts are needed to reach out-of-school youth, who may or may not have access to the Internet. Given the complexity of HIV preventive behavior, it is unlikely that a single intervention will affect HIV incident rates. Instead, an arsenal of prevention programs available through different modes and for different populations is needed.

### Implications

In an environment where HIV prevalence is high yet resources are limited, having an easily accessible and scalable program such as CyberSenga helps increase young people's access to information needed to reduce their risk for HIV infection. As the Internet becomes more affordable and, therefore, more widely accessible in Africa, CyberSenga and other Internet-based interventions are becoming increasingly viable [4]. Future research should include a replication of CyberSenga with an active control group; and in other Ugandan as well as greater East African settings to assess its impact on HIV incidence in less controlled environments.

## Supporting Information

Checklist S1 **CyberSenga CONSORT Figure Checklist.**
(DOC)

Protocol S1 **CyberSenga Study Research Protocol Approved by Ethics Review Boards.**
(DOC)
