# Supplementary material for: Adolescent Abstinence and Unprotected Sex in CyberSenga, an Internet-Based HIV Prevention Program: Randomized Clinical Trial of Efficacy
Source: PLoS One. 2013 Aug 14;8(8):e70083. doi: 10.1371/journal.pone.0070083 (PMC3743833; doi:10.1371/journal.pone.0070083)
Supplement: Protocol S1 — CyberSenga Study Research Protocol Approved by Ethics Review Boards. (DOC) [file pone.0070083.s002.doc]

**CyberSenga: Harnessing the power of the Internet to prevent HIV in Ugandan Youth**

Michele Ybarra, Ph.D. Principal Investigator, Internet Solutions for Kids, Inc.

Julius Kiwanuka M.D. Co-Principal Investigator, Mbarara University School of Technology

Protocol last updated: October 5, 2011

**A. Background and hypothesis**

HIV/AIDS is a major contributor to morbidity and mortality in Uganda (UNAIDS, 2005). Despite years of consistent decline in incidence rates (USAID, 2002), recent data suggest rising incidence (Shafer et al., 2006; Kamali et al., 2002). Further, among young people between the ages of 15 and 24 years who engaged in higher-risk sex in the previous year, 38% of men and 56% of women did not use a condom (UNAIDS, 2005). Perhaps most telling, surveys suggest that HIV knowledge is on the decline among young people in Uganda compared to their same-aged counterparts surveyed in 1990 (UNAIDS, 2005).

New and innovative methods are needed to affect behavior change and promote HIV risk reduction strategies among young people. The Internet is a promising mode of intervention delivery in resource-limited settings because the costs associated with scaling up are minimal: dissemination of online material is the same if one person or 100,000 people use the program (Ybarra & Eaton, 2005). Just as important, it provides access to imperative health information in a stigma-free, anonymous atmosphere.

Our recent data published in PLoS indicate that 45% of adolescents in Mbarara, Uganda (a small municipality four hours outside of Kampala) have used the Internet, 78% of whom went online in the previous week (Ybarra et al., 2006). Already, one-third (35%) have looked for information about HIV/AIDS on a computer or the Internet. These data suggest that Internet-delivered adolescent risk reduction interventions are both desired and feasible in Uganda.

We will achieve the following objectives through this work:

**Objective 1:** Design a 6-hour, Internet-based HIV prevention program for adolescents. Content will be culturally tailored to the HIV preventive information, motivation, and behavioral skills needs of Ugandan adolescents.

**Objective 2:** Test the intervention in a randomized controlled trial (n=500) among adolescents attending grades Secondary 1-4 (similar to US high school grades 8^th^ – 11^th^) at day schools in Mbarara.

The intervention program design will be focused on promoting HIV preventive behaviors. Acknowledging that adolescence is a time of sexual debut for a large minority of youth (UNAIDS, 2005), we use a harm reduction approach, reflected by our main outcome, which is abstaining from unprotected sex during the 6-months post-intervention. We also recognize that abstinence from sex is the most effective method of HIV prevention, and therefore include as a secondary outcome measure, sexual abstinence during the 6-months post-intervention.

In addition to the training session about how to use the intervention program, the Internet-based intervention will be a 6-hour course.

Topics covered. Content will be written to address the important characteristics affecting HIV preventive behavior as identified in our theoretical model and confirmed in the qualitative study: HIV prevention information, motivation, and behavioral skills. Anticipated intervention topics are informed by the adolescent focus groups conducted in Musaka, Uganda by Muyinda et al (Muyinda et al., 2003). Content will acknowledge social-contextual factors that affect the sexual behavior of young Africans (Kinsman et al., 2001), specifically: 1) a high value placed on pre-marital sexual activity; 2) peer pressure plays a large role as do social norms; 3) financial and material transactions are often involved in sexual intercourse; 4) although condom usage has increased, there remains frequent unprotected sex; and 5) there continues to be a gap between knowledge and continued risk behavior. We also expect issues about gender inequality, poverty, stigma, violence, coercion, and family functioning to emerge from the qualitative study findings. Figure 1 shows how these locally-identified topics map to our theoretical model.

**HIV prevention motivation**

Attitudes

Peer pressure

Norms

Family planning

Value placed on pre-marital sex

Intentions

**Figure 1: Theoretical model and associated content area**

***HIV prevention behavior:***

*Use of condoms during sex*

**HIV prevention information**

HIV/AIDS education

STD education

Role of substance use in HIV (e.g., decreased inhibition)

**HIV prevention behavioral skills**

HIV testing and treatment seeking for STDs

Talking about sex

The role the women plays in partner communication (clear communication)

The role the man plays in unwanted sex (coercive sex)

Condom use and locally applicable information (i.e., where to obtain condoms in Mbarara)

Techniques for avoiding unwanted/risky sex

Delaying first sex

Partner reduction

**Interactive vignettes**

***Possible cultural factors:***

*Gender inequality*

*Poverty*

*Stigma*

*Violence*

*Coercion*

*Local access to condoms*

Based upon sex-specific norms and cultural beliefs, some modules will be tailored by sex (e.g., family planning, traditional sex practices). Culturally sensitive information and vignettes will be the backbone of the intervention, with the qualitative study findings driving the framework for the final content.

Content and interactive features. Given the unique cultural aspects of HIV education and behavior in Uganda, it is important that the intervention be developed within the community as opposed to modified from a Western program. We believe that the culturally appropriate, community-based intervention conducted by Muyinda et al (Muyinda et al., 2003) may provide a helpful framework from which to begin. Their use of surrogate sengas as narrators of intervention information and education was reportedly accepted not only by adolescent women but also adolescent men. Results of the study suggest increased HIV preventive behavior for both sexes in the intervention community versus control community.

Personal “guides” have been successfully used in the YouthNet project. Because the modern sengas were so resonant with young people in the earlier research (Muyinda et al., 2003; Muyinda et al., 2004), we propose to use this framework by including virtual sengas as ‘guides’ through the intervention. As a personalized character within the online course, the senga will introduce each topical area and then narrate the program as the topic is further elucidated. Communication strategies and behavioral skills will be reinforced through vignettes played out by role models matched to the user by age and sex. Important aspects of the vignette will be punctuated both by text and by the senga’s narration as was done successfully in YouthNet. We will confirm the saliency of the virtual senga as well as explore other possible narrators that young people find relevant and compelling among both adolescent women and men during the focus groups and Youth Advisory Council module testing.

Program exercises will be informed by Fisher et al’s (Fisher et al., 2002) Information-Motivation-Behavior skills HIV risk reduction intervention developed for youth, which has demonstrated HIV behavior change across many different groups of young people (Fisher et al., 1994; Fisher et al., 2002; Bryan et al., 2000; Linn.J.G. et al., 2001; Kalichman, 2005). Module 1 will focus on teaching correct **information** about HIV transmission and prevention, paying special attention to correcting common misperceptions identified during the qualitative study. Module 2 will focus on promoting **motivation** to change behavior by targeting norms and attitudes among adolescents in Mbarara illuminated in the qualitative study that lead to risky behavior. For example, depending on the qualitative study findings, we may examine peer pressure to have sex, financial and material transactions that can accompany sex, and the value placed on pre-martial sex among adolescents in Mbarara. Module 3 will reinforce motivation for behavior change through videos and cartoons. Module 4 will focus on the teaching of **behavioral skills** necessary for condom use and abstinence. Module 5 will focus on communication skills and teaching youth how to negotiate safer sex scenarios. Module 6 will interactively review the information, motivation and behavioral skills learned in the previous 5 modules.

**B. Study procedure and methodologies**

To develop the HIV prevention web site, we will use a mix of qualitative and quantitative methods.

Our first step in intervention design will be to conduct a qualitative study to examine the applicability of the IMB Model to HIV preventive behavior among youth in Uganda. Integrating lessons learned from the formative data, the program then will be developed with direct feedback from adults and youth in the community. The program will be tested in an ongoing fashion during the design phase to ensure proper execution of lessons learned during the formative testing. Finally, the intervention and protocol will be tested in a randomized control trial of 500 adolescents attending one of the five secondary schools in Mbarara.

### 1. One-on-one interviews (n=15 adults, 48 youth):

### We will use a qualitative approach to examine the applicability of the IMB model to the study of HIV-preventive behavior among youth in Uganda.

### First, individual semi-structured interviews will be conducted with 15 key adult informants who are “experts” on adolescent behavior and local culture. We will ask for their thoughts on adolescents’ ideas about HIV transmission, the motivations youth may have for engaging in unprotected and protected sex, and the behavioral skills young people have to engage in HIV risk reduction behaviors. Information gathered will be used to identify and refine concepts to be explored later in interviews with adolescents (see Appendix A).

### Following the heuristic schema (Ware et al., 2006), the IMB model will be examined for goodness of fit by answering the following analytic questions:

1. Are the IMB model’s basic concepts accurate for the study of HIV-preventive behavior among youth in the Ugandan setting? (Are all relevant basic concepts represented appropriately?)
2. Are concepts important for this setting represented in the model?
3. Are the meanings of the model’s basic concepts (the ways they are specified or operationalized) accurate for the study of HIV-preventive behavior among youth in the Ugandan setting?
4. How well does the model capture the complexity of HIV-preventive behavior among youth in the Ugandan setting?

To answer these questions, semi- structured individual interviews will be conducted with 48 adolescents (see Appendix B). Interviews will be conducted in Ruyenkole by Ugandan research assistants trained in qualitative interviewing techniques and translated into English post-hoc. With permission, interviews will be audio taped using digital recording technology. Findings will be analyzed by Dr. Ware and examined by the research team for intervention design implications, culminating in a ‘content map’ for each of the six modules to be used as a script for discussion with the Community Advisory Board and Youth Advisory Committee.

2. Quantitative survey (1,496):

We will conduct a quantitative survey with a representative number of students across the five partner schools to confirm aspects of the IMB model, as well as to identify Popular Opinion Leaders who will recruited for the Youth Advisory Council (below). Power analyses indicate that 62 students need to be surveyed in each of the 4 classes at Sentah College, and 78 students in each of the 4 classes at each of the four larger schools (Mbarara Secondary, Mary Hill, Ntare School, and Mbarara High School).

3. Technology assessment (n=10):

To further help direct the training and introduction module of the prevention program, we will observe ten randomly identified adolescents using the Internet. Participants will be assigned several tasks (e.g., to open up Internet Explorer, to find three web sites that have information about HIV/AIDS, and to sign up for a free email account at Yahoo!). The focus of the exercise will be to identify challenges and deficits in knowledge among the adolescents in navigating the Internet, and how this may relate to their success or challenge in navigating the prevention web site.

4. Community Advisory Board (CAB) (n=20).

Twenty adult volunteers from the community will serve on the CAB and provide ongoing feedback during the curriculum development phase. Before the drafting of each module, we will meet with the CAB and present our proposed content areas and elicit feedback about advised ways to discuss these issues, specific content that should be included, and culturally appropriate ways to discuss sensitive issues. These ideas will be integrated into the curriculum and shared with the CAB at the following meeting for further refinement.

5. Youth Advisory Council (n=20).

The Youth Advisory Council (YAC) will serve two roles, first as youth curriculum ‘experts’ and second as pilot testers of the actual modules. During the curriculum development phase, we will meet with the YAC before the drafting of each module. We will present the proposed curriculum topics and solicit ideas about specific information that should be included, suggested ways to discuss sensitive issues, and information about locally specific behaviors and skills that may be relevant (e.g., where the youth-friendly places to buy condoms are). This information will be integrated into the curriculum and shared with the YAC at the following meeting for further refinement.

To ensure that our modules are not just evidence-based but also engaging (Hoppe et al., 2004), the Youth Advisory Council will pre-test each of the modules once the curriculum is integrated into the web site design. When each module is completed, each YAC member will be asked to come to the Computer Lab and complete the module. We will watch how they navigate the module to identify any technical challenges, and conduct a one-on-one structured interview afterwards to identify aspects that the youth liked as well aspects that could be modified or improved. Because we are committed to reducing stigma associated with HIV, our study materials will be non-judgmental and written with a harm-reduction approach. To identify unanticipated stigma that may result from study materials, we will query the YAC about their reactions to the module characters, asking questions that tap into stigmatizing perceptions (e.g., whether they believe the characters are ‘safe’ to hang out with; alternatively, whether the characters should be ostracized).

6. Methodology pilot (n=96)

The logistics of the randomized controlled trial will be complicated and difficult. To ensure that we are properly trained for the task, and that the schools have appropriate expectations of what we need to be successful when we are in field, we are proposing to add a Methodology Pilot. We will recruit, randomize, and follow 96 students (24 in each school) through a randomized controlled trial of MoodGYM, a five-lesson Internet program aimed to change thoughts and behaviors to improve depressive symptomatology. Half will be randomly assigned to the internet program; half will be assigned to the comparison group. We will focus on solving logistical challenges relevant to the randomized controlled trial including challenges involved in transferring a large number of secondary students from school to the Study cafe multiple times per week, adhering to a well scripted schedule that takes into account our Study cafe capacity and school activities, and examining whether students are willing to complete a multi-week Internet program. Also, we will be conducting the baseline and follow up surveys online to confirm that data can be validly collected in this mode.

Participants in the comparison group will be asked to come to the study office two times. The first time, they will complete a survey on the computer. Three weeks later, they will return to the study office and complete a second survey. Participants in the MoodGYM group will be asked to come to the study office seven times over three weeks. The first time, they will complete a survey on the computer. They will complete one MoodGYM lesson for the next five visits, each. On the seventh visit, they will complete the second survey. All students will complete the same surveys. Questions will include things about their feelings including depression, their experience with computers and cell phones, and also things about their relationships with friends and boyfriends or girlfriends. Students assigned to the MoodGYM program also will answer questions about their experience with the Internet program.

7. Randomized controlled trial (n=400):

Once the Internet-based program has been developed, we will test it in an intent-to-treat, missing=failure randomized controlled trial among 400 randomly identified adolescents in Mbarara. Subjects meeting inclusion criteria will be randomized to either the intervention (n=200) or the control (n=200) study group. Participants in the Intervention group will complete intervention modules aimed at increasing HIV preventive behavior. They will come to the study café once a week to complete a one-hour module each session. Participants in the control group will be a treatment-as-usual group; they will receive the sexual health education available at their schools. All children will participate in data collection surveys at three different time points: at baseline, the beginning of school Term II, and the beginning of school Term III.

To gather feedback about and understand the experiences with the CyberSenga program, we will conduct six focus groups with intervention participants after the final quantitative follow-up data collection period. Participants will be randomly identified from among the intervention participants. Groups will be stratified by biological sex, and grade (senior 2 versus senior 3 and 4).

**C. Research participant information**

1. Inclusion criteria:

Specific **inclusion criteria for youth include**:

1. Attendance at a partner secondary school
2. Enrollment in grades Secondary 2 through 4
3. Adult informed consent and adolescent informed assent
4. Use of a computer at least once in the past year (starting with the Methodology Pilot)
5. Not intending to vote in 2011 election (RCT only)

Participants are required to have some experience with computers so that they are able to successfully navigate through the Internet intervention. People who are voting in the 2011 election must travel back to their home in order to place their vote; these students will miss a week of the intervention. As such, students intending to vote will not be eligible for the RCT.

2. Exclusion criteria:

No other inclusion or exclusion criteria, including prior Internet use, will be applied. This is to ensure a wide variability of Internet expertise to enlighten the web site’s utility across a range of users.

1. Recruitment procedures:

Recruitment for the adult one-on-one interviews (n=15) with key adult informants who are “experts” on adolescent behavior and local culture will use a snowball design. This convenience sample of individuals who work with adolescents in the community will be identified through referrals of people who work with adolescents that have relationships with research staff. Participants will include (but not necessarily be limited to) physicians, nurses, HIV counselors, and teachers.

# Five secondary schools in Mbarara with which the study team has existing collaborative relationships established will be the sites where all youth study subjects will be recruited (see Table 1). The total sample pool is 3,630.

| **Table 1. Participating school characteristics** | | | | | | |
| --- | --- | --- | --- | --- | --- | --- |
| School characteristics | | A | B | C | D | E |
|  | Eligible enrollment (grades S1-S4) | 376 | 1006 | 551 | 976 | 721 |
|  | Ownership | Private | Government | Government | Government | Private |
|  | Religion | Muslim | None | None | None | Catholic |
|  | Male:Female ratio | 49:51 | 100:0 | 55:45 | 100:0 | 0:100 |
|  | | | | | | |

# Participants in the youth one-on-one interviews (n=48) will be randomly selected from Sentah College (A above) and Mbarara secondary school (C above). Specifically 6 youth in each of the 4 grades in both schools will be selected. Youth participants in the quantitative survey (n=1496), technology assessment (n=20), the Methodology pilot (n=96) and the randomized controlled trial (n=500) will be recruited randomly from each of the five study schools.

# Starting with the Methodology pilot, we will be working with 4 of the 5 schools. School E will not be included in either the Methodology pilot or the RCT. Because of constraints imposed by the school schedule, Secondary 1 students will not be included in the RCT.

Each headmaster will provide consent for our Project Coordinator to view a list of all current students who are in grades Secondary 1 - Secondary 4 (Secondary 2 – Secondary 4 for the RCT) and therefore eligible to participate in the study. The list will be sorted by class in alphabetical order. Using a random numbers list, study candidates will be selected randomly from each class in each school.

Following the procedures used with success by Ybarra and colleagues in previous research involving secondary school students in the same schools in Mbarara, we will recruit adolescent interviewees in the following manner. In each of the two participating schools, teachers in each of secondary grades 1-4 (or secondary grades 2-4 for the RCT) will introduce the study briefly and identify those students randomly chosen to participate. S/he will ask those students to assemble to meet with the Ugandan study interviewer at the next break. At that time, the interviewer will describe the study in more detail and determine eligibility.

Starting with the Methodology pilot, participants will need to have used the computer at least once in the past year. To determine eligibility, students will complete a brief Eligibility Screener. We will over-sample in order to screen enough students to identify the targeted number eligible participants for each research activity.

The Screener will be brief but include several questions so that it will not be clear which question is the ‘eligibility question’ to which one should say ‘yes’ to in order to be eligible. In other words, to increase the veracity of self-report, the eligibility criteria will not be made clear in the screener.

We will review the screeners and contact those who are eligible to participate to see if they are interested in taking part in the program.

Those students who evidence interest and live locally will be given an information sheet describing the study and a consent form for their parent or legal guardian to sign indicating consent for the youth to participate.

For boarding students or orphans, school principals will provide consent.

Youth who agree and have adult consent to participate will also provide written informed assent.

Recruitment for the Youth Advisory Council (n=20) will based upon peer-nomination so that they represent the opinion leaders of the target population. We will conduct a brief paper-and-pencil survey in Year 2 to measure the information, motivation, and behavioral skills associated with HIV preventive behavior among all youth in the target schools. Included in this survey will be one question about the student in their grade that youth look up to the most. The one student mentioned the most frequently in each of the four secondary grades within each of the five partner schools will be invited to take part in the YAC. If they decline, we will invite the second most frequently nominated student and so forth. If two or more students are equally popular, we will randomly choose one of the students to recruit.

Recruitment for the Community Advisory Board (n=20) will be based upon referrals from participants in the adult one-on-one interviews and people who work with adolescents that have relationships with research staff.

1. Expected number of individuals to be enrolled (+age, sex, ethnicity)

Below is the expected number of participants for the research project.

| Table 2. Expected number of individuals to be enrolled | | | |
| --- | --- | --- | --- |
| Research activity | n | Estimated length of involvement | Estimated place in project timeline |
| Individual interviews with adults | 15 | 1 hour | Year 1 |
| Individual interviews with adolescents | 48 | 1 hour | Year 1 |
| Quantitative survey | 1,496 | 1 hour | Year 2 |
| Technology assessment | 10 | 1-2 hours | Year 3 |
| Youth Advisory Council | 20 | 6-12 hours | Year 2-3 |
| Community Advisory Board | 20 | 6 hours | Year 2-3 |
| Methodology Pilot (MoodGYM) | 96 | 2 hours for control, 7 for the intervention | Year 3 |
| Randomized controlled trial | 400 | 4 hours for control, 10 hours for intervention | Year 4 |

We expect 100% of youth participants to be African. Sixty percent are expected to be male, although this is more a reflection of the schools (i.e., 2 are all-male; see Table 2) than of the recruitment strategy per se. In mixed-sex schools, we anticipate 50% of recruited participants will be female. The expected age range is between 12-18 years of age, although given our inclusion criteria based upon secondary grades 2-4, 80% of participants are expected to be between the ages of 14-17.

4. Randomization

##### Participants in the Methodology pilot and the randomized controlled trial will be randomized to a control or intervention group. Participants will be automatically randomized immediately following the baseline survey. The two study arms will be balanced by youth biological sex and self-reported sexual activity in the past 24 months. The RAs will receive the treatment assignment for each participant from a password-protected Internet web site maintained by the study biostatistician. The RAs will not have access to the participants’ baseline data and therefore will not know the participants’ responses to any of the questions, including sexual behavior.

5. Remuneration to participants:

Participants will not incur costs associated with transport because it will provided to them by the study.

Because cash incentives are not culturally appropriate in Uganda, especially for children, we offered free Internet or cell phone time (2000 shillings) to participants in the early stages of the research. Neither incentive was particularly popular with participants. Internet use was particularly challenging because students' time off campus (e.g., to come to the study cafe to use the Internet) was tightly controlled. Given that cell phones were not allowed in many of the boarding schools, offering free minutes was not consistent with school policies. In some cases, we enhanced the incentive options with chocolate or other candy. Starting with the Methodology pilot, we will not provide any type of incentive.

6. Informed consent process

Adult participants: Ms. Wyatt and/or the local study representative [To Be Hired] will obtain written consent at the outset of the interview session. Consent will be obtained following the staff meeting or at a convenient time agreed upon by the potential participant and researcher. Clinic staff will be reminded they are completely free to take part in this research or not, and that their decision will have no impact on any benefits or services to which they are entitled, or on the circumstances of their employment. Clinic staff consent forms will be presented in English.

Youth participants:

School Principal consent to access adolescents students: School Principals will serve as adult proxies for parents and provide written consent for research personnel to access a list of student names, and for research personnel to contact eligible students. Students will be randomly chosen for study recruitment.

Parental consent for student participants: Parents of students that are randomly chosen to participate in the study will be asked to provide written informed consent (see below) for their child to participate in the study. Students will be given an enrollment packet that includes a cover letter explaining the study to the parents, parent consent form, and youth assent form. Parents who are willing to allow their child participate will sign the form and give it to their child to bring to school and give to the research assistant. Two consent forms will be provided to each parent of randomly selected child: one in English and the other in Runyankole, the local language. To ensure families do not feel coerced to participate, they will be assured both in the consent form and by research professionals at the community meetings that participation is voluntary. Participation or non-participation will not affect their child’s school standing or any future medical care. School principals will serve as adult proxies and provide informed consent for youth who are orphans or are boarding students.

Adolescent informed assent: Student participants will provide written informed assent. The informed assent process will be carried out in a private location by a trained study representative in the study participant’s language of choice at the time of study enrollment. A trained research assistant will sit with the candidate while he or she reads the assent form. The research assistant will verbally highlight the important aspects of the document. Youth will be reminded they are completely free to choose to take part in the research or not, and that their decision will not affect their school standing.

7. Potential risks

First, it is possible that participants may be ridiculed or otherwise be stigmatized for participating in the study because of its HIV focus. We are taking several measures to counteract this by fully educating the public, the schools, and the students about the purpose of the study and the importance of HIV prevention information. Adults and youth in the community will have a direct voice in the content development. We also are monitoring the potential for stigma in study materials by pre-testing all modules with our Youth Advisory Council, asking specifically about whether each module has promoted in any way stigmatizing beliefs. Because of the familiarity with HIV prevention programs, we believe that these efforts will minimize the potential for stigma. Further, the innovative nature of the program, including Internet and cell phone-based incentives, may even influence young people to see participants as ‘cool’.

Second, the data may be viewed by people who are not designated to view the information (e.g., a peer looking over their shoulder). We will create a Study Internet café with private study carrels to help prevent this. Participants who access the intervention at JM Internet café will do so at a dedicated study carol that is private.

Third, to ensure the privacy of data, all data will be transmitted securely using SSL (TLS) 128-bit encryption across the Internet (HTTP). SSL provides users with the assurance of access to a valid, "non-spoofed" site, and prevents data interception or tampering with sensitive information. We have chosen to obtain the majority of the information through the web site to increase the confidentiality of the reports. The SSL certificate that will be used for this project will use 128-bit encryption, the preferred security level of government and financial institutions. 128-bit encryption offers protection that is virtually unbreakable. For example, if a hacker could crack a standard 40-bit SSL session in a day, it is estimated that it would take well beyond a trillion years to accomplish the same thing against a 128-bit SSL session. To ensure against the remote possibility that an intruder gains access to stored data, all data stored will be further encrypted, making use of any acquired data nearly impossible. Dedicated servers eliminate security issues involved with shared hosting environments where hundreds of web sites and users reside on one shared web server as well as ensuring both physical and network security. Quantitative data and the intervention web site will be located on a dedicated server at Omnis Network's secure collocation facilities located in Torrance, CA. Omnis Network provides a world class, award winning collocation infrastructure with high-speed Cisco-powered Tier-1 Internet backbones and security services. The dedicated server will be configured with redundant hard drive array (RAID) to ensure reliability by protecting from hard drive failure. Data will be backed up to DAT tape or external hard drive(s) with weekly archiving off site to protect from fire/natural disaster. Qualitative data recorded on video cassettes and tape recorders will be secured in locked cabinets at the UCSF study site in Mbarara. Only the Project Director will have the key to access the cabinets.

Fourth, with the increase in Internet use, concerns about increased exposure to pornography online and its potential negative effect on youth development have been raised. Although recent data suggest that these concerns may not necessarily be borne out by the data (Ybarra & Mitchell, 2005), we nonetheless will install blocking software on all Study lab computers to ensure a safe and secure Internet experience for all participants. All Internet cafes in town have a strict ‘no pornography’ rule, ensuring that children will not be exposed when redeeming their free Internet time either.

8. Potential benefits

If the intervention has an impact, it will increase the HIV preventive behaviors enacted by adolescents, thereby reducing their risk for contracting HIV. All participants will receive HIV prevention information published by The AIDS Study Taskforce (TASO) at follow-up. Once developed and tested, this program will be a powerful tool to add to the arsenal of HIV preventive programs available to young people in resource-limited settings. This research project will develop the first-of-its-kind Internet-based HIV prevention program for adolescents in Uganda. Once efficacy is demonstrated, future studies will focus on evaluating the effectiveness of the intervention in less controlled environments.

9. Plan for confidentiality of data:

Chesapeake IRB, the OHRP-approved IRB designated as Internet Solutions for Kids’ IRB of record, and the local IRB at Mbarara University, along with collaborator IRBs will review all data collection instruments and procedures. Quantitative data collected by the researchers will be data entered if collected via pen and paper, or automatically stored in secure servers if collected online. Data will be stored on either ISK’s secure server, or that of Vovici or Survey Monkey, international online survey companies. In both cases, data will be password protected. Paper-based data (e.g., qualitative data) will be stored in locked cabinets at the secure UCSF research study office on the campus of Mbarara University and at Dr. Norma Ware’s offices at the Harvard Medical School, Boston, MA.

In order to assure privacy for adolescents and to minimize risk, we will be using computer-based data collection procedures as much as possible. This reduces the number of people who view the data, and increases self-disclosure on sensitive topics. Access to the data will be password protected.

Each participant will be given a random unique identifier in the data set stripped of all personal information to protect confidentiality. Data sets used for analysis will contain project identification numbers but neither names nor other identifying information such as home address. Identification information will be retained by Internet Solutions for Kids, Inc. for the duration of the study and stored separately from the responses provided by subjects.

Collaborators will receive data stripped of personal identifiers. To ensure complete confidentiality, access to the ‘key’ linking personal identifiers to usernames and passwords will be restricted to the Principal Investigator. Reports will not identify individual adults or youth. Dr. Ybarra will oversee the data storage and reporting procedures. Upon completion of the analysis of the data collected in this study, computerized data files will be erased and paper records shredded. Reports will only use aggregated data. No report of individual data will be provided. The Core research team will have weekly meetings utilizing real-time Internet technology to monitor the progress of the study.

10.
